# The short- and long-term effects of resistance training with different stability requirements

**DOI:** 10.1371/journal.pone.0214302

**Published:** 2019-04-01

**Authors:** Atle Hole Saeterbakken, Alexander Olsen, David George Behm, Hilde Bremseth Bardstu, Vidar Andersen

**Affiliations:** 1 Western Norway University, Faculty of Education, Arts and Sports, Department of Sport, Food and Natural Sciens, Sogndal, Sogn og Fjordane, Norway; 2 School of Human Kinetics and Recreation, Memorial University of Newfoundland, St. John's, Newfoundland, Canada; University of Belgrade, SERBIA

## Abstract

The aim of this study was to assess the short- and long-term effects of resistance training (RT) with different stability requirements. Fifty-nine men underwent a 3-week familiarization period followed by a 7-week training period. During familiarization, all participants trained four sessions of squats with a Smith machine, free weights and free weights standing on a wobble board. After week-3, participants were randomized into a low (Smith machine), medium (Free-weight) or high (Wobble board) stability RT program, and Control group. All participants were tested pre-, after week-3 and post-intervention. Ten repetition maximum (10RM), rate of force development (RFD), electromyography (EMG) and maximum voluntary isometric contraction (MVIC) were tested in all three squat conditions in addition to countermovement jump (CMJ) on stable and unstable surfaces, and muscle thickness. After familiarization, greater 10RM loads (21.8–27.3%), MVIC (7.4–13.5%), RFD (29.7–43.8%) and CMJ (4.9–8.5%) were observed in all conditions. Between week 3 and 10, the Free-weight and Wobble board groups similarly improved 10RM in all conditions. Smith machine group demonstrated greater improvement in the trained exercise than the medium and high stability exercises. All training groups showed similar improvement in muscle thickness, RFD and MVIC. There was no CMJ improvement on the stable surface, but the Wobble board group demonstrated significantly greater improvement on the unstable surface. In conclusion, low, medium or high stability RT resulted in similar improvements in trained and non-trained testing conditions except for greater CMJ on the unstable surface in the Wobble group. Greater 10RM strength in trained than non-trained exercise was only observed in low stability group. Familiarization was associated with substantial improvements in 10RM and CMJ, with greater improvement associated with higher stability requirements. These findings suggest that high stability can increase strength, muscle thickness and explosive measurements similar to training with lower stability.

## Introduction

Task specificity or training specificity in resistance training leads to greater strength gains when the tests and training involve similar tasks [1–3]. However, there is substantially lower transferability of strength to dissimilar tasks, despite the involvement of similar muscle groups (i.e., squat and leg extension) [1–3]. Task specificity in resistance training is dependent on the velocity [4], contraction form [1, 2] and movement pattern [5].

Proponents of instability in resistance training have suggested that unstable conditions might improve coordination, proprioception, balance and muscle activation to a greater extent than stable conditions [6–8]. The most frequently used approaches to increase stability requirements attempt to increase the degrees of freedom, such as using free weights instead of training machines [5, 9] or changing from a stable to an unstable surface [5, 10]. It has been argued that under unstable conditions, the muscles involved will prioritize stability over force production [6, 8]. Studies examining the acute effects of instability have demonstrated decreased dynamic strength [11], force output [12, 13], rate of force development (RFD) [12] and jump height [14] when unstable surfaces are used compared to stable surfaces. This has led several researchers to argue that instability in resistance may provide a substantially lower stimulus for strength-training adaptions compared to traditional approaches [8, 15, 16].

Studies examining instability in resistance training have not provided comprehensive or conclusive evidence regarding muscle activation or the transfer of tasks to more stable conditions [11, 15, 17, 18]. The lack of comprehensive evidence may be attributed due to several limitations in previous studies. For example, minimal familiarization to unstable conditions before the experimental test may favour tasks performed in stable conditions [10, 19]. Differences in training status among the participants (i.e. untrained vs. athletes) may result in different conclusions examining a similar task [5, 19, 20]. In addition, the use of absolute rather than relative intensity examining different stability requirements may explain the inconclusive results [11, 18, 21].

A systematic meta-analysis [22] of 22 training studies reported that unstable compared with stable resistance training demonstrated inconsistent results. Some studies reported training-induced changes in favour of unstable resistance training while others show greater training benefits with stable resistance training [22]. The authors concluded that unstable resistance training had limited additional benefits for muscle strength, power and balance. Furthermore, the use of unstable versus stable resistance training is only partially recommended. Still, no studies have examined the long-term effect of instability resistance training on morphological adaptions.

Only a handful of intervention studies have examined resistance training programs with different stability requirements [3, 5, 10, 19, 23]. In general, these studies report similar strength improvement [10, 19, 23, 24], and similar electromyographic (EMG) activity regardless of the stability requirements [5, 10]. For example, inexperienced resistance trained participants trained for 7 weeks either under stable or unstable resistance training conditions, demonstrating no overall differences (i.e. leg extension strength, static and dynamic balance, long jump, shuttle run, and sprint) except for greater training adaptations for number of sit-ups performed (8.9%) and right leg hopping test (6.2%) [25]. Sparkes and Behm [10] reported that the unstable training group tended (p = 0.06) to show greater improvement in stable-to-unstable force ratio than the stable group. However, these authors only examined two levels of stability requirement [10].

More recently, Saeterbakken et al. [5] compared the task specificity and the time-course of adaptations of athletes undertaking chest press training using either a Smith machine, dumbbells on a bench or barbell bench press on a Swiss ball. The groups training with the unstable Swiss ball and dumbbells demonstrated greater improvement with the trained exercise than the stable Smith machine group. The greatest improvements were observed within the first 3 weeks for all groups, but were most notable for the two unstable training groups [5]. In addition, Saeaterbakken et al. [5] examined the transferability of strength to a non-trained exercise (traditional free-weight bench press). The transferability of strength was similar for the stable Smith machine group compared to the overall improvement in trained exercises, but lower for the Swiss ball group and dumbbell group.

Despite growing interest in the effect of instability in resistance training, the majority of the scientific literature has not examined long-term adaptations, and most have tested only a few parameters and not compared the transferability of strength between different stability requirements [11, 13, 24]. Therefore, the purpose of the study was two-fold: (1) to examine short-term adaptations of EMG and muscle properties (muscle thickness, muscle strength and explosive measurements) following a 3-week familiarization period in participants training with three squat exercises across three different stability levels; (2) to examine the long-term adaptions on muscle properties following a 7- week progressive resistance training program with either low, medium or high stability requirement. We hypothesized that 1) during the familiarization period the greatest improvement would be observed in the exercises with greatest stability requirement and 2) in the intervention period, greater strength and explosive measurements would be observed for the groups training under the medium- and high-stability conditions [5].

## Methods

### Design

**Fig 1:**
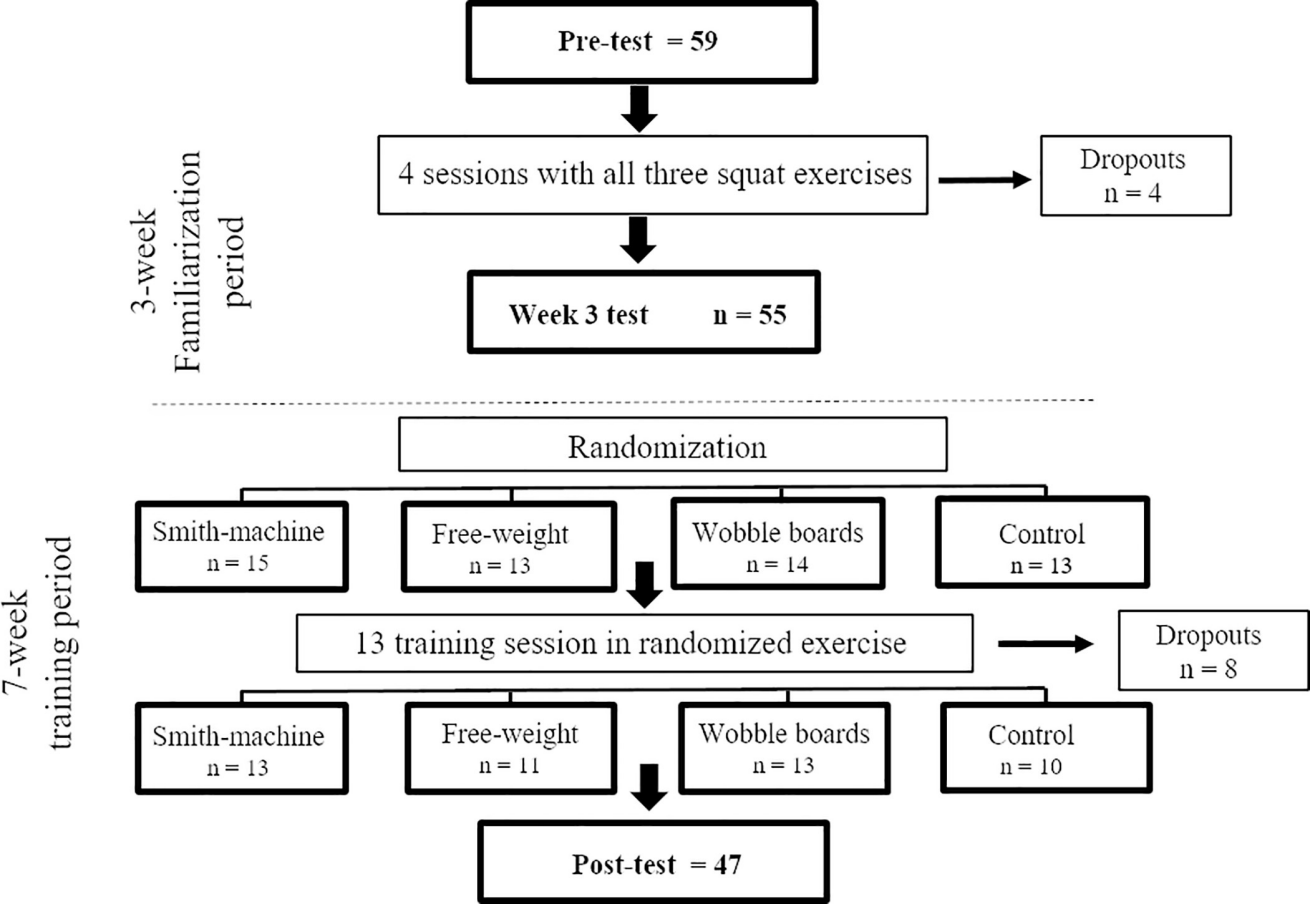
An overview of the study.

A randomized, controlled study was used to examine the effects of three squatting exercises with different stability requirements by assessing 10 repetitions maximum (10RM), maximal voluntary isometric contraction (MVIC), RFD, countermovement jump (CMJ), muscle thickness and EMG activity. The three exercises, performed with either a low, medium or high degree of instability, involved squats performed using a Smith machine (low), squats with free weights (medium) or squats with free weights while standing on two wobble boards (high). All participants were tested three times: prior to the intervention (pre-test), after 3 weeks (post-familiarization), and after 10 weeks (7 weeks post-familiarization) of training (Fig 1). During the first 3 weeks (familiarization period), all participants trained each of the three squat exercises. After the week 3-test, the participants were randomized into either a control group or groups that performed training twice per week for 7 weeks with either a low, medium or high degree of instability. Participants in the Control group refrained from all resistance training targeting the legs between week 3-test and week 10 test (post-test).

### Participants

Fifty-nine physical active and healthy males volunteered as participants (age 22.7 ± 3.3 years, body weight 78.9 ± 8.4 kg, height 180.2 ± 5.5 cm). The participants had on average 4.7 ± 3.8 years of strength training experience (Table 1), but none had performed leg strength training regularly for the past six months (<1 session per week). The inclusion criteria to participate in the study were no regular leg strength training in the past six months, not being able to lift twice their body weight in the squat, perform a 90° squat with proper technique (see testing procedures) and free of injuries or pain in addition to being male.

**Table 1:** An overview of the anthropometric data and years with resistance training.

	SM# (n = 13)	FW# (n = 11)	WB# (n = 13)	CON# (n = 10)
Age (years)	23.2 ± 4.8	23.0 ± 1.8	23.6 ± 3.7	21.1 ± 1.7
Body-weight (kg)	75.9 ± 7.2	81.5 ± 9.3	78.9 ± 7.7	81.2 ± 10.9
Height (cm)	178.4 ± 7.2	182.9 ± 4.1	180.2 ± 4.6	179.8 ± 7.4
Years of resistance training	4.7 ± 4.4	4.7 ± 4.1	6.1 ± 3.5	4.4 ± 4.5

# SM = Smith machine group, FW = Free weight group, WB = Free weight standing on wobble boards group and CON = control group.

### Ethics statement

All participants were informed orally and in writing of the study procedures and the possible risks. Informed written consent was obtained from the participants before inclusion in the study. The study was approved by the Regional Committees for Medical Health and Research Ethics in Norway (39024/3/HIT) and conformed to the Helsinki Declaration (2013). Participant gave his written informed consent (in accordance with PLOS consent guidelines) for his images to be reproduced in this manuscript.

### Testing procedures

Before each testing and training session, the participants performed a standardized warm-up of free-weight squats consisting of 20 repetitions of 25% of 1RM, 10 repetitions of 50% of 1RM and eight repetitions of 70% of 1RM [5]. Self-reported 1RM was used for the warm-up loads before the pre-test. After the pre-test, the highest load lifted in training was used. After the week 3 test, the warm-up was performed according to the squat modality of the group (low, medium or high instability) to which the participants were randomized.

All tests were conducted during one session in which measurements of muscle thickness, MVIC, RFD, 10RM in each of the three squat variations, and CMJ on stable and unstable surfaces were performed. Muscle thickness was always measured first, but the remaining tests were randomized to avoid bias in performance due to fatigue. The test order for each participant was performed identically as the pre-test.

### Muscle thickness

The thickness of the participants’ vastus lateralis muscle (dominant foot) was measured using ultrasound (LogicScan 128 EXT-1Z; Telemed, Vilnius, Lithuania) before starting the warm-up procedure, and results were analyzed with the corresponding software (Echo Wave II; Telemed, Vilnius, Lithuania). The participants were instructed to lay supine in a relaxed position (small pillow under knee) with the knees extended at approximately 170°. The thickness was measured half-way between the greater trochanter and lateral condyle [26]. Muscle thickness was determined from six measurements of the distance between the deep and superficial aponeurosis [26]. The lowest and highest values were excluded, and the mean of the four remaining measurements was used in further analyses [26]. The coefficient of variation (CV) of the muscle thickness was 1.1%.

### MVIC, RFD AND EMG

**Fig 2:**
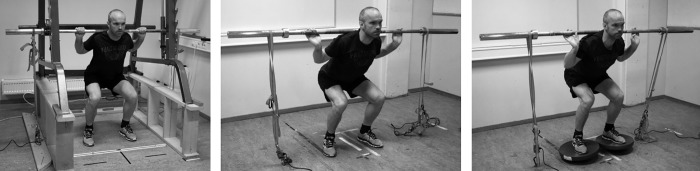
Testing procedures for the RFD, EMG and MVIC with low stability requirement, medium stability requirement and high stability requirement.

The MVIC, EMG and RFD measurements were performed during the same test. The participants stood with a natural sway in their lower back (lumbar curve), self-selected foot distance, and their knees at a 90° angle, which was measured manually along the femur and fibula with a protractor. Two force cells (Ergotest Technology AS, Langesund, Norge) were attached to the floor, and two adjustable non-elastic bands were attached between the force cells and barbell for squats using the Smith machine, free weights or free weights combined with wobble boards (Fig 2). The non-elastic bands were adjusted so that the knee angle remained at 90° during the test. The height from the barbell to the floor was measured for each condition for use in week 3 and post-test to ensure identical conditions. There were no differences in barbell height (measured from the barbell to the foot) between the conditions. The participants were not allowed to change their knee angle before beginning the test, and were instructed to press the barbell lightly upwards to tighten the bands between the barbell and the force cells. The participants then generated maximum force as quickly as possible and maintained this maximal force production for at least 3 s [13]. Three attempts were conducted in each of the three squat modalities with a 2–3 min rest between each attempt and between each exercise.

The electrodes were placed on the vastus lateralis (VL), vastus medialis (VM), rectus femoris (RF), biceps femoris (BF) and soleus (SOL) of the dominant foot according to current recommendations, SENIAM guidelines and previous studies [13, 27]. Anatomical landmarks were used to ensure equal placement of the electrodes, and the same test leader conducted all EMG measurements. Body hair at each of the locations was shaved, then skin was abraded and washed with alcohol before placing the gel-coated surface electrodes (Dri-Stick Silver circular sEMG Elektroder AE-131; NeuroDyne Medical, Cambridge, MA, USA). To minimize noise from external sources, the raw EMG signal was filtered and amplified using a pre-amplifier located as close as possible to the pickup point. The signals were filtered through high- and low-pass filters (maximum cutoff frequency of 8–600 Hz). The raw EMG signals were converted to root-mean-square (RMS) signals using a hardware circuit network (mean constant of 12 ms, frequency response 450 kHz, total error ± 0.5%). The RMS-converted signal was sampled at a rate of 1000 Hz using a 16-bit A/D converter with a common rejection rate of 106 dB. The EMG was normalized using the highest muscle activation over a 3 seconds window. The participants performed two maximal voluntary isometric contraction for all muscles using manual resistance. They were instructed to obtained maximal force as quickly as possible [28]. For the biceps femoris, the participants lay in a prone position with a knee angle of approximately 45° before trying to perform a flexion of the knee. For the quadriceps, the participants sat on a bench with a 90° knee angle before trying to extend the knee. For the soleus, the participants sat in a chair with a 30° flexion in the ankle before trying to extend it. All exercises have been described in details elsewhere [12, 28, 29].

The MVIC and EMG measurements overlapped, and the beginning and the end of the isometric force outputs were identified using the MuscleLab 6000 system (Ergotest Technology AS, Langesund, Norge). The MVIC was calculated as the highest mean force output over a 2.0 second window with the least variation in force, and the EMG activity was calculated over the same window [13]. The RFD was calculated from the onset of force production over a 0.2 second window [30]. The attempt with the highest MVIC and RFD was used in further analyses. The MVIC, EMG and RFD data were analyzed with the corresponding software (v10.5; Ergotest Technology AS, Langesund, Norge).

### Ten repetition maximum (10RM)

The participants performed squats with a low, medium or high degree of instability using a Smith machine (low), free weights (medium) or free weights while standing on two wobble boards (high). The order of the tests was randomized. The squat depth was measured and controlled by placing a horizontal band behind the participants. They were instructed to touch the band lightly with their gluts before pressing upwards [31]. The height of the band corresponded to a 90° angle of the knees. The participants used their preferred foot width. The distance between the heels and big toes of feet were measured and used in testing and training [31]. The participants maintained a natural sway in their lower back during the entire lift. The loads were lowered at a controlled speed before being elevated again. Only small pauses were allowed between repetitions. Belt and knee wraps were not allowed. If the participants completed 10 repetitions, the loads were increased until failure or until the participants and test leader agreed that the load was the true 10RM. Between 1–3 attempts were used to identify the 10RM loads. The participants were allowed 3–5 min rest between each attempt and between the three squat modalities [5, 31].

### Countermovement jump (CMJ)

The CMJ exercise was performed on a stable (force platform) and unstable surface (two wobble boards). The two wobble boards (Theraquatics, Montgomery, USA) were placed on the force platform (Ergotest Technology AS, Langesund, Norge) used to measure the jump height. The wobble boards were identical to those used to perform squats with a high degree of instability. The CMJ began from a standing position with hands placed at the hip. The participants used a self-selected depth [26]. The jump height was calculated by the impulse using a commercial software program (MuscleLab V8.13; Ergotest Technology AS). Three attempts were performed for both the stable and unstable CMJ, with 1–2 min rest between each attempt. The highest jump for each condition was used in further analyses.

### Training procedures

The participants were instructed to refrain from all lower-body strength training apart from the training conducted in the study. Between the pre-intervention and week 3 tests, all participants conducted four squat sessions separated by 3 days. In the sessions, the participants conducted one series of 10RM in each of the three squat modalities. The order was randomized. If the participants were confident they could increase the load after completing a set, the load was increased before the next session. The squat depth was measured and controlled for each participant as described in the 10RM testing procedure [31]. Two test leaders acted as spotters and gave verbal encouragement during testing and training. Four participants dropped out of the study during the familiarization period. Three to five days after completion of the fourth training session, the participants were re-tested (week 3 test; see Fig 1).

The warm-up procedures were identical to the testing procedures, but were performed differently according to the group they were randomized into (e.g., the Smith machine group used the Smith machine). The group specific training was performed twice a week for 6 weeks (12 sessions). The training was performed with a linear increase in intensity; sessions 1 to 4 were conducted with three series of 10RM, sessions 5 to 10 with four series of 8RM, and sessions 11 and 12 with four series of 6RM. If the participants were able to complete the last set with the correct number of repetitions, the load was increased by 2.5kg or 5.0 kg in the next session [5, 31]. The test leaders gave oral encouragement, acted as spotters and ensured proper technique. The test leaders were present during 99.6% of all sessions. In the 10th week (session 13), the participants performed a session identical to that performed during the familiarization period (10RM in all three squat modalities). The session was conducted to increase the 10RM reliability in the post-intervention test conducted 3–5 days after session 13.

Between the week 3 and post-intervention tests, eight participants dropped out for reasons unrelated to the study (Fig 1). There was no difference in training volume between the training groups (p = 0.256).

### Statistics

The Mauchly`s test of sphericity indicated that the assumption of sphericity had not been violated, x^2^ (2) = 1.292–5.713, p = 0.057–0.524). For data collected during the familiarization period (pre-intervention to week 3 tests), two-way ANOVA (3 levels of stability requirements × 2 testing times) was used for analysis of the variables 10RM, MVIC, RFD, CMJ and EMG. The same dependent variables were compared between the week 3-test and post-intervention test in a two-way mixed design ANOVA (4 groups × 2 testing times) for each of the three stability requirements using SPSS software (v23.0; Chicago, IL, USA). When differences were detected with ANOVA, paired t-tests with Bonferroni post hoc correction were applied. Statistical significance was accepted at p ≤ 0.05, and all results are presented as the mean ± standard deviation and Cohen’s effect size (ES). An ES of 0.2 was considered small, 0.5 medium and 0.8 large [32].

## Results

### Familiarization period (pre-intervention to week 3)

There was no significant interaction between exercise and time for 10RM loads, MVIC, RFD or EMG activity (F = 0.019–1.106, p-values between 0.334 and 0.981). For 10RM loads, there was a main effect for time (F = 891.344, p ≤ 0.001) and main effect for level of stability requirements (F = 12.124, p ≤ 0.001). The highly unstable (wobble board) exercise demonstrated greater relative improvements than the medium (free-weight) and low (Smith machine) stable exercises (Table 2). The results for MVIC and EMG activity in the soleus and vastus lateralis showed a significant increase over time (F = 3.930–22.059, p-values ranging from ≤0.001 to 0.050), but not exercise (F = 0.121–1.462, p-values between 0.236 and 0.886). For RFD and the other muscle measures, there were no significant effects of time or exercise (F = 0.130–3.549, p-values between 0.062 and 0.878, Table 3). For details about 10RM loads, MVIC, CMJ and EMG, see Table 2, Table 3, S1 File, S2 File and S4 File.

**Table 2:** Changes in 10RM, MVIC, RFD and CMJ for the different stability requirements between pre- and week 3 test.

	Stability requirements	Pre-test	Week 3 test	% improvement	p-values	Effect size
10RM# (kg)	Low (SM#)	109.1 ± 21.1	130.9 ± 18.5	22.2 ± 14.6%	p<0.001	1.10
	Medium (FW#)	101.4 ± 17.2	122.0 ± 16.7	21.8 ± 12.7%	p<0.001	1.22
	High (WB#)	91.1 ± 16.5	122.0 ± 16.7	27.3* ± 13.0%	p<0.001	1.86
MVIC# (N)	Low (SM)	887 ± 271	955 ± 245	10.2 ± 36.9%	p = 0.067	0.26
	Medium (FW)	874 ± 226	932 ± 252	7.4 ± 19.4%	p = 0.144	0.24
	High (WB)	854 ± 243	958 ± 232	13.5 ± 29.0%	p<0.001	0.44
RFD# (Ns^-1^)	Low (SM)	2403 ± 1070	2661 ± 1080	43.8 ± 120.5%	p>0.407	0.24
	Medium (FW)	2517 ± 1166	2450 ± 1273	39.2 ± 150.8%	p>0.407	0.05
	High (WB)	2446 ± 1090	2707 ± 1276	29.7 ± 103.1%	p>0.407	0.22
CMJ# (cm)	Stable surface	34.4 ± 4.7	35.9 ± 4.5	4.9 ± 8.8%	p<0.001	0.33
	Unstable Surface	28.6 ± 5.0	30.8 ± 4.8	8.5* ± 11.0%	p<0.001	0.45

* relatively greater improvements than the other exercises p<0.05.

# 10RM = 10 repetition maximum, MVIC = Maximal voluntary isometric contraction, RFD = Rate of force development, CMJ = countermovement jump, SM = Smith Machine, FW = Free-weight, WB = Wobble boards.

**Table 3:** Relative (%) changes in electromyographic (EMG) activity between pre-test and week 3 test for the different stability requirements.

	Muscle	Low stability requirement	Medium stability requirement	High stability requirement
EMG#	Vastus medialis	15.3 ± 53.8 ES# = 0.01	30.5 ± 73.5% ES = 0.33	26.8 ± 64.6% ES = 0.23
	Vastus lateralis	14.3 ± 53.9% ES = 0.20	17.7 ± 57.0% ES = 0.24	15.6 ± 38.5% ES = 0.34
	Rectus femoris	11.9 ± 48.7% ES = 0.04	14.6 ± 55.9% ES = 0.18	18.0 ± 59.1% ES = 0.28
	Soleus	36.2 ± 99.7% ES = 0.31	56.7 ± 51.6% * ES = 0.45	44.6 ± 101.8% * ES = 0.52
	Biceps femoris	6.9 ± 46.5% ES = 0.11	8.5 ± 37.2% ES = 0.20	49.7 ± 199.1% ES = 0.34

* Greater EMG activity at week 3 test p<0.05.

# ES = effect size, EMG = electromyography.

### Training period (week 3 to post-intervention)

#### Ten repetition maximum (10RM)

**Fig 3:**
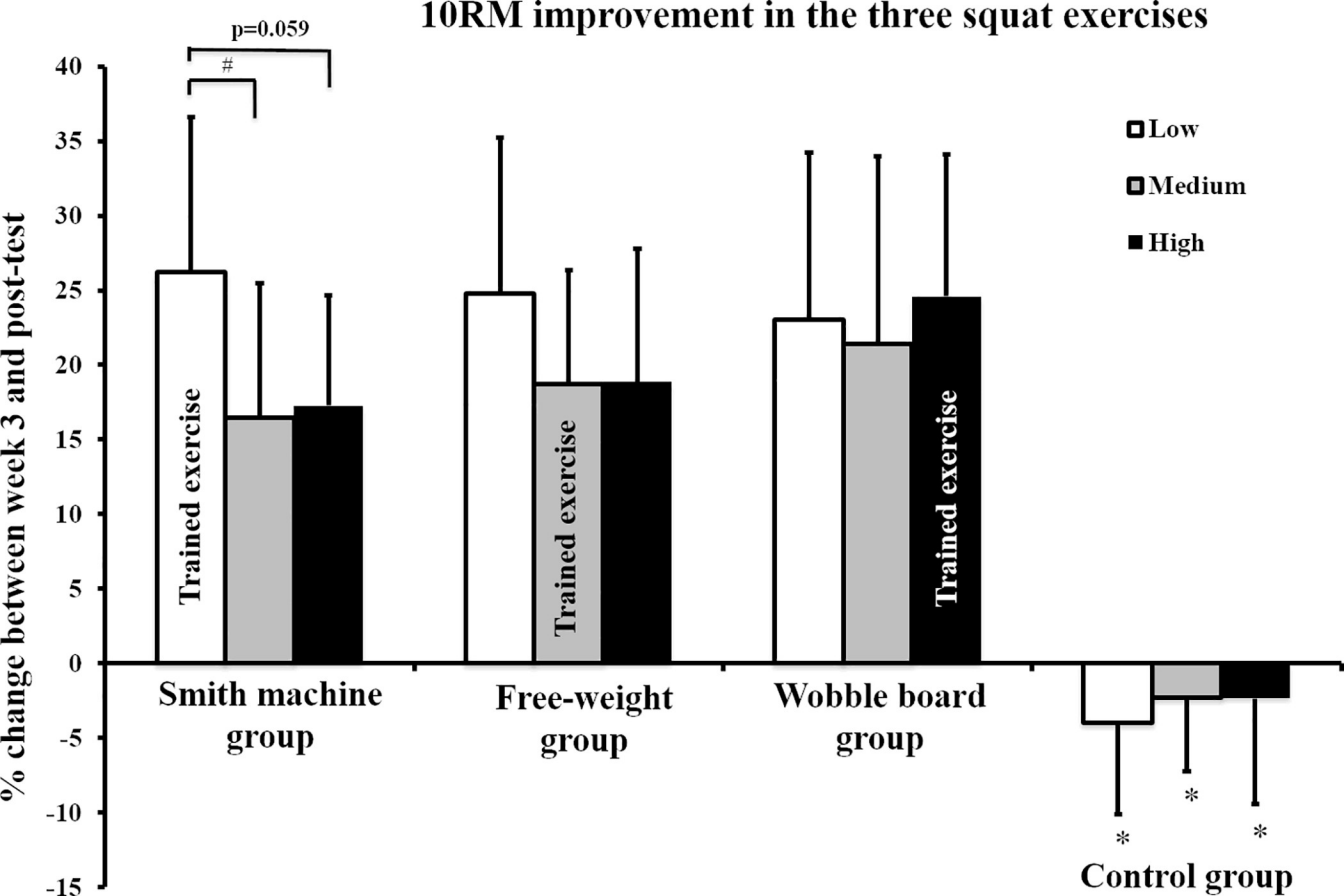
Improvement in 10RM loads in trained and non-trained exercises.

There was a significant interaction between time and group for all three exercises (F = 15.904–26.199, p ≤ 0.001). The Smith machine group demonstrated greater improvement in trained exercise than free-weight exercise (p = 0.044, ES = 0.14), and wobble board exercises (p = 0.060, ES = 0.30, Fig 3 and Table 4). The Free-weight–and Wobble board groups demonstrated similar improvement in 10RM strength in trained and non-trained exercises in the post-intervention test (p ≤ 0.001, ES = 0.14–1.24). Still, all training groups demonstrated greater improvements than the control group (p ≤ 0.001, ES = 2.32–3.92). All details are presented in Table 4 and Fig 3.

**Table 4:** Changes in 10RM, MVIC and RFD with low, medium or high stability requirements for the groups between week 3-test and post-test.

		Low stability requirement		Medium stability requirement		High stability requirement		
		Week 3-test	Post-test	Week 3-test	Post-test	Week 3-test	Post-test	ES#
10RM# (kg)	SM#	127.7 ± 19.9	160.4 ± 22.7*	116.4 ± 18.5	144.2 ± 19.7*	110.0 ± 16.4	134.6 ± 18.8*	1.41–1.61
	FW#	133.4 ± 19.6	154.5 ± 20.3*	125.9 ± 18.5	148.9 ± 19.9*	116.9 ± 15.7	140.0 ± 19.1*	1.08–1.46
	WB#	132.5 ± 17.9	155.4 ± 23.3*	125.2 ± 16.0	148.9 ± 22.7*	116.9 ± 14.2	145.8 ± 21.7*	1.14–1.62
	CON#	130.3 ± 1825	124.5 ± 14.6	121.0 ± 12.9	118.3 ± 14.5	114.3 ± 15.7	112.3 ± 21.4	0.11–0.37
MVIC# (N)	SM	941 ± 251	1063 ± 285*	920 ± 265	1062 ± 328*	972 ± 256	1047 ± 297	0.27–0.48
	FW	1012 ± 270	1131 ± 241	943 ± 280	1133 ± 277*	977 ± 241	1090 ± 227	0.40–0.48
	WB	977 ± 284	1135 ± 272*	1008 ± 269	1114 ± 256*	1019 ± 237	1135 ± 273*	0.40–0.57
	CON	902 ± 339	941 ± 356	873 ± 337	916 ± 320	889 ± 335	911 ± 343	0.10–0.22
RFD# (Ns^-1^)	SM	2530 ± 697	3461 ± 1073*	2710 ± 1191	3145 ± 1160	2926 ± 1035	3009 ± 1401	0.07–1.03
	FW	2412 ± 1445	3695 ± 1081*	1913 ± 1330	3463 ± 1083*	2651 ± 1456	3387 ± 1418	0.51–1.28
	WB	3113 ± 1288	3565 ± 1000*	3003 ± 1403	3565 ± 987	3025 ± 1506	3265 ± 1579	0.18–0.46
	CON	2553 ± 604	2653 ± 1025	1938 ± 896	2501 ± 1209	2129 ± 1071	2217 ± 1381	0.07–0.53

* greater than week 3-test.

# ES = Effect size. 10RM = 10 repetition maximum, MVIC = Maximal voluntary isometric contraction, RFD = Rate of force development, SM = Smith machine group, FW = Free-weight group, WB = Wobble board group and CON = Control group.

#### Maximal voluntary isometric contraction (MVIC)

There was no significant interaction nor a main effect of group on MVIC (F = 0.568–1.255, p-values between 0.302 and 0.639), but there was a significant main effect for time for all stability requirements (Smith machine, free-weight and wobble boards (F = 8.083–19.056, p-values from ≤0.001 to 0.007). None of the groups demonstrated greater improvement in trained exercise than non-trained (task specificity), but the Smith machine and Wobble board groups showed significant improvements of strength to non-trained stability requirement (p < 0.050). All details are presented in Table 4.

#### Rate of force development (RFD)

There was no significant interaction or main effect of group on RFD (F = 0.275–1.994, p-values from 0.230 to 0.843), but we observed a main effect for time for the exercises with low and medium stability requirements (Smith machine and Free-weight; F = 15.930–18.832, p ≤ 0.001), but not for the high stability requirement (Wobble board; F = 1.489, p = 0.230). All training groups demonstrated improvement in RFD in the Smith machine (lowest stability requirements (p = 0.022–0.041; ES = 0.53–1.03). In the free-weight exercise (medium stability requirement), none of the training groups improved RFD (p = 0.062–0.445; ES = 0.37–0.66). The control demonstrated no improvement in the Smith machine or free-weight exercise (p = 0.105 and 1.000, ES = 0.05 and 0.76). All details are presented in Table 4.

#### Muscle thickness

There was an interaction between time and group for muscle thickness (F = 4.521, p = 0.008). The Smith machine, Free-weight and Wobble board groups showed 6.3%, 3.8% and 4.4% increases in muscle thickness, respectively (p-values ranging from <0.001 to 0.049, ES = 0.14–0.45), but none of the groups demonstrated greater improvement over any other group (p-values from 0.826 to 1.000). The Control group had a -0.1% decrease in muscle thickness (p = 0.327, S3 File).

#### Countermovement jump (CMJ)

**Fig 4:**
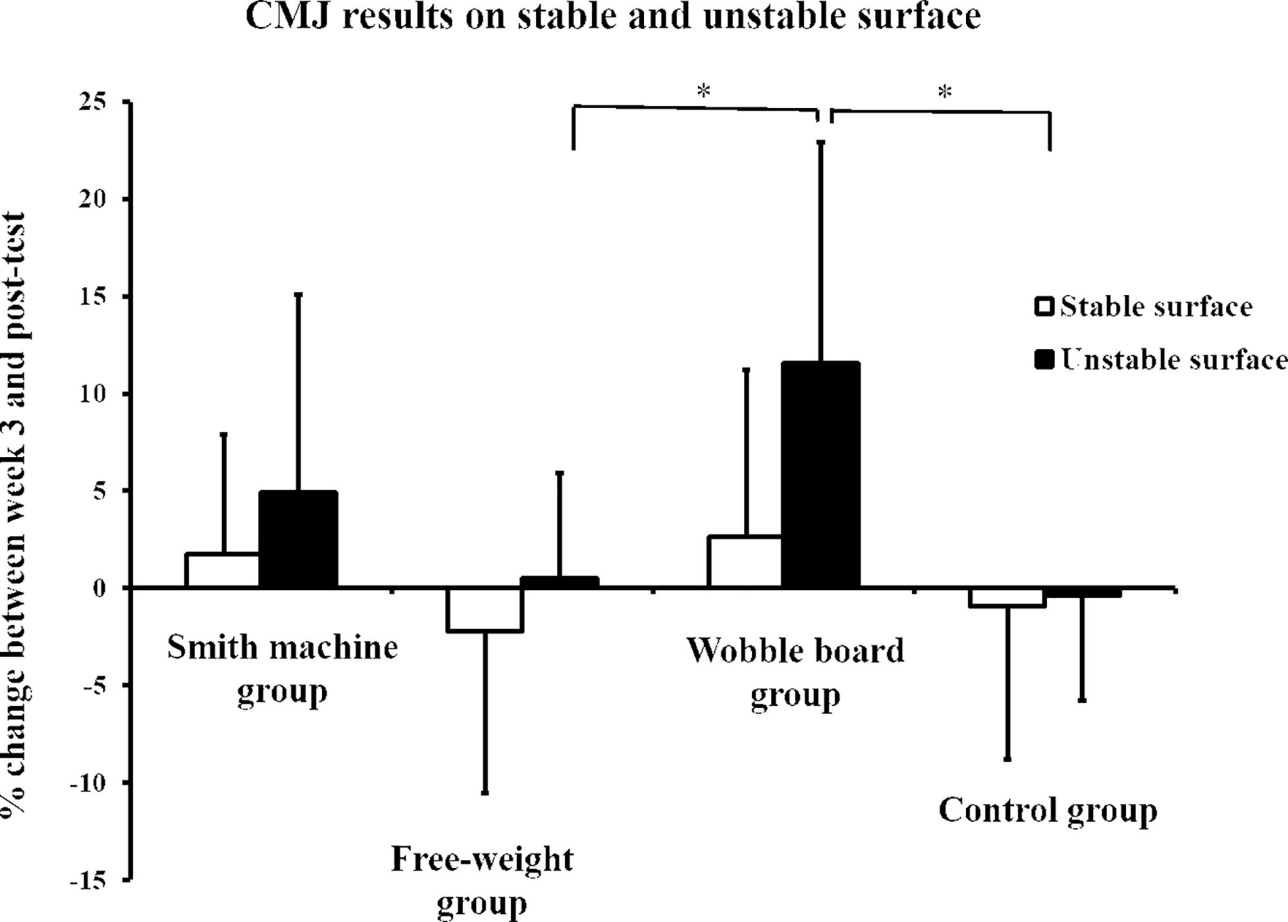
Improvement in CMJ on stable and unstable surfaces.

There was an interaction observed for CMJ on an unstable surface (F = 4.304, p = 0.010), but there were no interactions or significant effects observed for the stable surface (F = 0.046–1.416, p-values between 0.251 and 0.831). The Wobble board group was the only group to demonstrate an improvement on the unstable surface, and this improvement was greater than both the Free-weight and Control groups (p-values from 0.014 to 0.020, ES = 0.66 and 1.36). All details are presented in Table 5 and Fig 4.

**Table 5:** Jump height for the groups at week 3 and post-test.

	SM# group		FW# group		WB# group		CON# group	
	Week 3-test	Post-test	Week 3-test	Post-test	Week 3-test	Post-test	Week 3-test	Post-test
CMJ stable surface (cm)	36.9 ± 4.6	37.4 ± 3.9	35.1 ± 4.9	34.4 ± 5.9	36.8 ± 4.8	37.6 ± 4.7	34.4 ± 3.4	34.1 ± 4.7
Effect size	0.12		-0.13		0.17		-0.07	
CMJ unstable surface (cm)	32.0 ± 4.1	33.4 ± 3.6‡	30.8 ± 6.7	30.8 ± 6.2	30.9 ± 4.5	34.1 ± 3.5*‡	29.1 ± 3.6	29.0 ± 4.1
Effect size	0.36		0.00		0.79		-0.03	

*greater than week 3 test

‡ greater than CON.

# SM = Smith machine group, FW = Free-weight group, WB = Wobble board group, CON = Control group, CMJ = countermovement jump.

#### Electromyographic activity (EMG)

With the low stability requirement, there was a significant main effect of group on the EMG activity of the rectus femoris (F = 3.200, p = 0.034) and a significant main effect of time on the soleus (F = 6.571, p = 0.015). The post hoc analysis demonstrated no significant differences between the groups in rectus femoris (p = 1.000, ES = 0.00–0.59), but 22% (±21.8%) greater soleus activation in the Free-weight group (p = 0.015, ES = 0.31). For the other muscles, there was no interaction nor significant effects on EMG activity (F = 0.052–2.544, p-values from 0.071 to 0.869). With the medium stability requirement, there was an interaction for the vastus medialis (F = 2.899, p = 0.047) and a significant main effect of group on the EMG activity of the rectus femoris (F = 2.900, p = 0.047). Post hoc analysis for both muscles showed no significant difference (p-values between 0.056 and 1.000). For the other muscles, there were no interactions or significant main effects on EMG activity (F = 0.065–3.281, p-values between 0.078 and 0.834). No interactions or significant main effects were observed for the high stability requirement (F = 0.300–3.211, p-values between 0.082 and 0.933).

## Discussion

The familiarization period (pre–week 3-test) led to improvements in 10RM loads (all exercises) and CMJ (stable and unstable surfaces), with greater improvement observed with the high stability requirement than other stability requirements. During the intervention period (week 3 –post-test), all training group improved 10RM strength in all three conditions (Smith machine, free-weights and wobble board). Only the Smith machine group demonstrated greater 10RM improvement in the trained exercise than the other tested exercises (task specificity). The Free-weight group and Wobble board group demonstrated similar strength to the non-trained exercise (transferability of strength between conditions). For MVIC and RFD, no group demonstrated greater improvement in the trained exercise than the other conditions. However, the Wobble board group demonstrated greater improvement in CMJ on the unstable surface than the Free-weight and Control groups.

### Familiarization period (between pre-test to week 3 test)

As hypothesized, the participants improved their 10RM loads in the three squat exercises from pre-intervention to week 3-test, with greater improvement observed for the exercises with the highest stability requirement (free weights on wobble boards). The familiarization period contained four training sessions consisting of only one set of 10RM in each of the three exercises. Despite this, the percentage improvement was similar to the improvement observed during the training period (7 weeks of training, twice per week, with 3–4 sets). These results demonstrate a massive short-term familiarization effect with instability, which was most likely caused by learning effects (improved inter-muscular coordination) to the different conditions [2]. The results are of significant importance and have great methodological implications. Results from previous studies that did not include familiarization sessions prior to comparing exercise with different stability requirements [15, 17, 33], should be interpreted with caution especially when patients or beginners to resistance training are included. However, Wahl and Behm demonstrated [34] no differences between conditions when resistance trained athletes performed exercises with different stability which may be a result of the participants’ training statues. Nevertheless, the results were supported by previous findings [5, 35]. For example, Saeterbakken et al. [5] examined different stability requirements in bench-press exercises, and demonstrated greatest improvement in the group with the greatest stability requirement during the first 3 weeks of training. The greater improvement in 10RM loads with the high stability requirement or learning effects may have resulted in greater improvement in CMJ under unstable conditions compared to stable conditions. These findings were supported by Kean et al. [36] who found improved CMJ height after 6 weeks of balance training (no resistance). The enhanced balance improving force vectors (more vertical and less horizontal force outputs) as well as decreased co-contractile activity [36].

Despite an improvement in 10RM strength by 22–27% following the familiarization period, there were no significant improvements in MVIC or RFD independent of stability requirements. The lack of differences may be explained by a lack of contraction specificity, short training period and similar EMG activity in the present study [2]. Similar EMG activity and the large variation in muscle activation (see Table 3) may be related to the training status of the participants [2, 37].

### Training period (week 3 to post-intervention test)

After the training period, all groups improved 10RM strength in all exercises and the improvements were greater than the Control group. The Free-weight and Wobble board groups demonstrated similar strength improvements between trained and non-trained exercises (i.e. transferability of strength), but the Smith machine group demonstrated greater improvement in the trained (Smith machine testing) than non-trained exercises (i.e. task specificity). The lack of free weight and wobble board group task specificity results were surprising. However, unstable resistance exercises tend to prioritize the stabilization functions in the muscles before force generation [6, 7]. The unstable exercise programs for the Free-weight and Wobble board groups may have promoted mobilizing functions letting the participants generate high external force while stabilizing the joints [5, 7, 10]. The group training with the lowest stability requirement (Smith machine group), could focus primarily on moving the barbell with minimum attention to stability and balance. However, when testing with medium and higher stability requirements, the muscles involved will prioritize stability over force production [6, 8]. The similar strength in trained and non-trained exercise in the Free-weight and Wobble board groups may have been a result of the familiarization period. The marked improvement in 10RM load after the familiarization period may have decreased the potential for further improvement (ceiling or plateau effect) or resulted in a short-term learning effect which made the different stability requirements manageable in the post-intervention test [5].

The 10RM results in the present study were not as hypothesized or supported by previous findings. Although, Sparkes and Behm [10] demonstrated similar improvement in dynamic strength, an improved stable-to-unstable force ratio for groups who trained on unstable surfaces compared to those who trained on stable surfaces was observed. Furthermore, Saeterbakken et al. [5] found that the training group with the lowest stability requirement was the only group that did not demonstrate greater improvement in trained exercise compared to bench press exercises with greater stability requirements. However, the study by Saeterbakken et al. [5] examined bench press in trained athletes, and, importantly, did not include a familiarization period, which may help to explain the different findings. The familiarization period may therefore explain the contradictory results compared to previous studies [5, 10]. Nevertheless, all training groups in the present study demonstrated similar improvements in 10RM strength, which were greater than the Control group.

The Wobble board group was the only group which demonstrated improvement in CMJ, but only when using the unstable surface. The improvement was greater than the Free-weight and Control groups. Based on the task specificity and previous speculation [7, 38], the result for the Wobble board group was as hypothesized. However, since the Smith machine group trained with the lowest stability requirements, they should theoretically show improvement in CMJ on the stable surface. Still, none of the groups demonstrated improvement in CMJ on the stable surface, meaning that the Wobble board group did not display transferability between stability requirements. The results are supported by previous findings. For example, Sparkes and Behm [10] demonstrated a tendency (p = 0.09) for greater improvement in CMJ for the unstable group compared to the stable group. However, previous studies were limited by only testing CMJ on a stable surface [10, 39].

None of the training groups demonstrated greater improvement in trained compared to non-trained exercises (task specificity) for the MVIC. The lack of task specificity for the trained exercise could be a result of similar training stimulus, confirmed by the similarities in training volume, muscle thickness and EMG activity between groups or the testing procedures. When testing MVIC with the highest stability requirement, the participants were able to stabilize the segments before generating their maximum force. However, an identical testing procedure employed in an acute study demonstrated greater MVIC in stable compared to three unstable exercises with increasing stability requirements [13]. The results were therefore surprising and not as hypothesized. Still, previous studies have demonstrated substantially lower improvement in isometric force than improvement from dynamic strength training [1, 2], which may explain the findings. It could be speculated that training during the familiarization period involving dynamic squats in similar conditions (high stability requirements), results in a short-term learning effect related to maximizing force generation while also maintaining stability [5]. The MVIC results of the present study were supported by previous studies [10, 39]. Both Sparkes and Behm [10] and Kibele et al. [39] demonstrated similar MVIC improvements following training on stable or unstable surfaces.

All groups demonstrated improvement in MVIC in trained exercises, but only the Wobble board group, demonstrated improvement in low and medium stability requirements. Based on these results, one could argue that training with the high stability requirement may favor force generation when compared to lower stability requirements, but not the opposite. The findings of Sparkes and Behm [10] partly support this speculation as they demonstrated a statistical tendency with a large magnitude effect size (p = 0.06, ES = 1.0) for improved stable-to-unstable MVIC force ratio to a greater extent in the unstable group than the stable group. The Free-weight group training with the medium stability requirements in the present study did not show improved low or high MVIC results.

None of the training groups demonstrated greater RFD improvement in trained exercise compared to the non-trained exercise. However, the Free-weight and Wobble board groups both showed similar improvement in RFD to exercises with lower stability requirements. To our knowledge, this is the first study to examine the effects of RFD in participants undertaking a strength training program with different stability requirements. The lack of greater improvement in trained exercise and differences between groups are likely due to the training conducted, as the participants lifted high loads with low movement velocity [4]. For example, the Wobble board group training with the highest stability requirement probably trained with lower velocity to maintain balance and stability. This could explain the lack of greater improvement in trained exercise which we hypothesized.

With the exception of soleus for the medium stability group, no changes in EMG activity were observed between groups or exercises even though all groups trained with a similar intensity and training volume. Based on the results, similar neuromuscular adaptations occur when different stability requirements are used. Similar findings have been reported in previous studies which compared the effects of different stability requirements [5, 10].

This is the first study to measure muscle thickness in a training intervention with different stability requirements. Previous studies have speculated that instability in resistance training may limit morphological adaptions, as acute studies show that a substantially lower load can be lifted in unstable exercises compared to stable resistance exercises [15, 17]. The evidence presented in the current study dismisses previous speculation by demonstrating similar improvement in muscle thickness between training groups. However, the present study might have been too short to detect significant changes, especially compared with the Control group. Nevertheless, previous studies have reported differences between groups within the same time frames [26]. Also, an overall low training volume of the legs might explain the similar results observed between the groups [30, 40]. However, there was no difference in training volume between the groups (weight lifted, repetitions and series) or squat depth [26], therefore, it would be surprising to have detected differences. Independent of the stability requirements, all training groups demonstrated an increase in muscle thickness.

The present study was limited by only including one exercise for the lower limbs. If several exercises had been included in the training program, differences in neuromuscular adaptation may have been evident between the groups. Secondly, with only 10–13 participants in each group there is an inherent risk that type II errors may be present. Further, the current study only included healthy and active men, therefore, the findings cannot be generalized to other populations or other training statues. Finally, the participants were encourage to maintain similar diet.

As a practical application, a healthy and active population have in general similar improvements and adaptions training with squats with different stability requirements. Still, training with high stability requirement (wobble board) resulted in greater jump height improvement under unstable conditions than the other conditions. Sports with an unstable surface (beach volleyball) may there benefit from training with instability. Importantly, there were no injuries or accidents related to lifting heavy loads with high stability requirement. Still, two spotters were present in all training supporting, helping and providing safety for the participants in their training routines. The authors can only hypothesis and encourage researchers to include patients or elderly, which we hypothesize may benefit to a greater extent to medium and high stability requirements in resistance training than the present population.

In conclusion, greater 10RM strength improvement in trained exercise and CMJ performed on an unstable surface was observed for the group with low (10RM) and high (CMJ) stability requirements. However, all training groups demonstrated similar improvements of 10RM strength, muscle thickness, CMJ on stable surface, MVIC and RFD regardless of whether training involved low, medium or high stability requirements. The four training sessions during the familiarization period led to substantial short-term improvement, with greater improvement observed with increasing stability requirements. These findings suggest that high stability requirements can increase muscle properties similar to training with lower stability requirements.

## Supporting information

S1 File Dataset. (XLSX)

S2 File Dataset. (XLSX)

S3 File Dataset. (XLSX)

S4 File Dataset. (XLSX)
